# Effect of oestrogen-dependent vasopressin on HPA axis in the median eminence of female rats

**DOI:** 10.1038/s41598-019-41714-z

**Published:** 2019-03-26

**Authors:** Kazuaki Nishimura, Kiyoshi Yoshino, Kenya Sanada, Hiroki Beppu, Yasuki Akiyama, Haruki Nishimura, Kentaro Tanaka, Satomi Sonoda, Hiromichi Ueno, Mitsuhiro Yoshimura, Takashi Maruyama, Hitoshi Ozawa, Yoichi Ueta

**Affiliations:** 1 Department of Physiology, School of Medicine, University of Occupational and Environmental Health, Kitakyushu, Japan; 2 Department of Obstetrics and Gynecology, School of Medicine, University of Occupational and Environmental Health, Kitakyushu, Japan; 3 Department of Anatomy and Neurobiology, Graduate School of Medicine, Nippon Medical School, Tokyo, Japan

## Abstract

The median eminence (ME) anatomically consists of external (eME) and internal (iME) layers. The hypothalamic neurosecretory cells terminate their axons in the eME and secrete their neurohormones regulating anterior pituitary hormone secretion involved in stress responses into the portal vein located in the eME. Magnocellular neurosecretory cells (MNCs) which produce arginine vasopressin (AVP) and oxytocin in the paraventricular (PVN) and supraoptic nuclei (SON) terminate their axons in the posterior pituitary gland (PP) through the iME. Here, we provide the first evidence that oestrogen modulates the dynamic changes in AVP levels in the eME axon terminals in female rats, using AVP-eGFP and AVP-DREADDs transgenic rats. Strong AVP-eGFP fluorescence in the eME was observed at all oestrus cycle stages in adult female rats but not in male transgenic rats. AVP-eGFP fluorescence in the eME was depleted after bilateral ovariectomy but re-appeared with high-dose 17β-oestradiol. AVP-eGFP fluorescence in the MNCs and PP did not change significantly in most treatments. Peripheral clozapine-N-oxide administration induced AVP-DREADDs neurone activation, causing a significant increase in plasma corticosterone levels in the transgenic rats. These results suggest that stress-induced activation of the hypothalamic-pituitary-adrenal axis may be caused by oestrogen-dependent upregulation of AVP in the eME of female rats.

## Introduction

The magnocellular neurosecretory cells (MNCs) which synthesise arginine vasopressin (AVP) and oxytocin (OXT) in the paraventricular (PVN) and supraoptic nuclei (SON) project their axons through the internal layer of the median eminence (iME) to terminations in the posterior pituitary gland (PP), from which AVP is secreted into systemic circulation^1^. On the other hand, AVP- and corticotrophin-releasing hormone (CRH)-containing neurones in the parvocellular neurosecretory cells (PNCs) of the PVN project their axons into the external layer of the ME (eME), from which the neurohormones are secreted into pituitary portal circulation^2,3^. Both AVP and CRH are known to stimulate adrenocorticotrophic hormone (ACTH) secretion from the anterior pituitary gland and corticosterone (cortisol) secretion from the adrenal gland^4,5^. The hypothalamic-pituitary-adrenal (HPA) axis is mediated not only through CRH^6–8^ but also AVP from PNCs. Sex differences in the HPA axis via CRH have been reported; however, to our knowledge, no study has examined sex differences in the HPA axis via AVP from PNCs.

The ME is a structure that joins the hypothalamus and pituitary gland and is divided into an internal layer and an external layer. The internal layer is a fibrous layer—the pathway of AVP and OXT neurones from the hypothalamus to the PP. The external layer is not an intermediate pathway from the hypothalamus but is where the axon terminates. Therefore, it is the site of information transmission to the pituitary portal vein and the area where the neural information is converted into humoral information. In order to communicate from the pituitary portal vein to the anterior pituitary gland, the eME also has the role of regulating the secretion of the anterior pituitary hormone. There are reports of age and long-term hormone treatment effects on the ultrastructural morphology of the ME^9^. The effect of sex differences and the oestrus cycle remains unknown, although several studies showed that AVP in the i/eME fluctuates after water deprivation^10^, salt loading^11^, and high-stress conditions^12^.

In this study, we aimed to investigate the sex differences in AVP dynamics using two types of original transgenic rats developed in our laboratory and to investigate the effect of AVP on the HPA axis. Our previously generated transgenic rats expressing the AVP-enhanced green fluorescent protein (eGFP) fusion gene^10–12^ and AVP-designer receptors exclusively activated by designer drugs (DREADDs) fusion gene^13^ were used in this study. Transgenic rats with the AVP-eGFP fusion gene allow visualisation of AVP expression and were thus used to investigate the dynamics of hypothalamic AVP by gender and oestrus cycle. The ligand of DREADDs is clozapine-N-oxide (CNO), and the receptor is human muscarinic acetylcholine receptor (hM3Dq)^14,15^. The AVP-DREADDs rat is a novel transgenic rat that is pharmacologically controllable and specifically expresses mCherry fluorescence in AVP neurones^13^. In some experiments, it was used to stimulate hypothalamic AVP neurones and investigate the function of AVP.

## Results

### AVP-eGFP fluorescence in the eME is stronger in females than males

**Figure 1:**
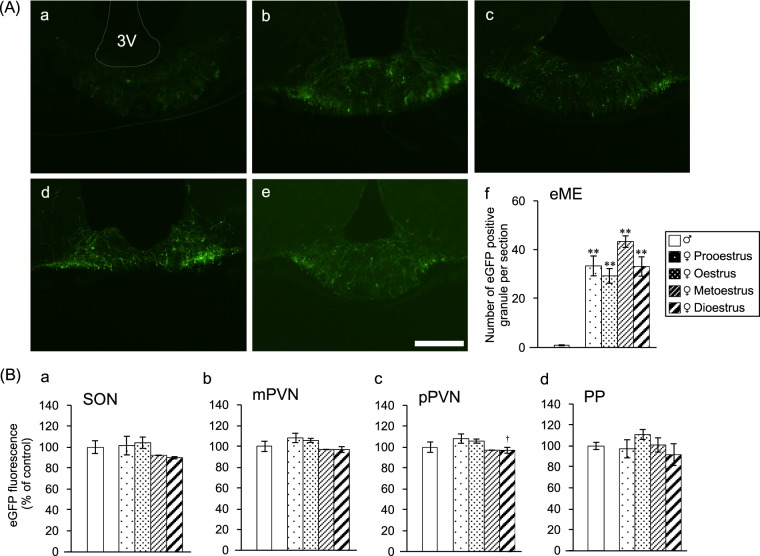
AVP-eGFP fluorescence in the eME is stronger in females than males. (**A**) AVP-eGFP-positive granules in the eME of (a) male, (b) prooestrus female, (c) oestrus female, (d) metoestrus female, and (e) dioestrus female AVG-eGFP transgenic rats. Scale bar indicates 100 μm. (f) The number of eGFP-positive granules in male rats and female rats during stages of the oestrus cycle [male (n = 5), prooestrus (n = 5), oestrus (n = 7), metoestrus (n = 4), and dioestrus (n = 6)]. The data were presented as the mean ± SEM (one-way ANOVA) (d.f. = 4, 22; F = 34.773; **P < 0.01 compared with male rats). (**B**) AVP expression of the (a) SON, (b) mPVN, (c) pPVN, and (d) PP. The data were presented as the mean ± SEM (one-way ANOVA) (d.f. = 4, 22; F = 4.053; ^†^P < 0.05 compared with prooestrus and dioestrus rats).

Examination of the hypothalamus of reproductive male and female AVP-eGFP transgenic rats revealed significant differences in the eME between males and females (group A experiment). Differences between the stages of the female oestrus cycle were also observed (Fig. 1(A)). The number of AVP-eGFP-positive granules in the eME of male rats was low (Fig. 1(A,a)), whereas female rats produced more AVP-eGFP-positive granules in the eME at all oestrus stages (Fig. 1(A),b–e). To distinguish the internal and external layers of the ME, a brain slice of an AVP-eGFP transgenic rat was labelled with an anti-OXT antibody whose secondary antibody exhibited red florescence. OXT passed through the internal layer and was not expressed in the eME. Therefore, the internal layer (pathway of OXT) showed red fluorescence, distinguishing it from the external layer (Supplementary Fig. 1a). The number of eGFP-positive granules based on the stages of the female oestrus cycle was higher in the prooestrus and metoestrus than in the oestrus and dioestrus stages (Fig. 1(A),f). The locations of the SON, magnocellular division of the PVN (mPVN), parvocellular division of the PVN (pPVN), and ME were determined according to the coordinates in the rat brain atlas of Paxinos and Watson^16^. AVP expression of the SON was not significantly different between males and females of all oestrus stages (Fig. 1(B),a). The SON was also labelled with the anti-OXT antibody to determine the localisation of AVP and OXT neurones (Supplementary Fig. 1b). AVP expression of the mPVN was not significantly different between males and females of all oestrus stages (Fig. 1(B),b). AVP expression of the pPVN showed a significant difference only in the prooestrus and dioestrus stages (Fig. 1(B),c). The mPVN and pPVN were labelled with the anti-OXT antibody to determine the localisation of AVP and OXT neurones. The whole PVN region showed green and red fluorescence, and the mPVN region showed circular green fluorescence (Supplementary Fig. 1c). There was no significant difference in the AVP-eGFP fluorescence of the PP between males and females of all oestrus stages (Fig. 1(B),d). These data suggested that the presence of AVP granules from PNCs in the eME is influenced by gender.

### Ovariectomy (OVX) diminishes AVP-eGFP fluorescence in the eME

**Figure 2:**
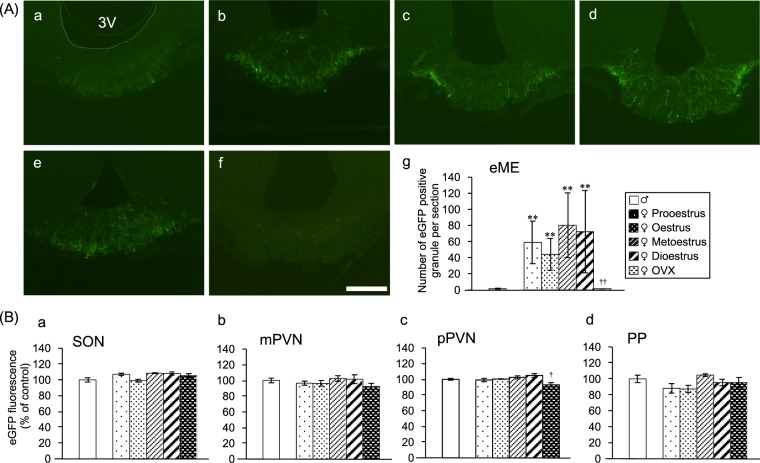
OVX diminishes AVP-eGFP fluorescence in the eME. (**A**) AVP-eGFP-positive granules in the eME of (a) sham-operated male rat, sham-operated female rats in the (b) prooestrus, (c) oestrus, (d) metoestrus, and (e) dioestrus period, and (f) OVX female rat. Scale bar indicates 100 μm. (g) The number of eGFP-positive granules in male rats, female rats during stages of the oestrus cycle, and OVX female rats [male (n = 5), prooestrus (n = 5), oestrus (n = 5), metoestrus (n = 4), dioestrus (n = 6), and OVX (n = 7)]. The data were presented as the mean ± SEM (one-way ANOVA) (d.f. = 5, 26; F = 51.314; **P < 0.01 compared with male rats, ^††^P < 0.01 compared with female oestrus and OVX rats). (**B**) AVP expression of the (a) SON, (b) mPVN, (c) pPVN, and (d) PP. The data were presented as the mean ± SEM (one-way ANOVA) (d.f. = 5, 26; F = 3.396; ^†^P < 0.05 compared with sham-operated male and OVX rats).

The change in the AVP granules of the eME in the hypothalamus was found to be due to sex differences. In order to investigate the influence of sex on AVP granules, we performed OVX, which created an oestrogen-deficient state (group B experiment). Male and female AVP-eGFP transgenic rats were used. Sham operation was performed in all male and female rats except those in the OVX group. In the previous experiment, AVP granules were confirmed in the eME in all female oestrus stages. Interestingly, AVP granules of the eME disappeared in the OVX group; AVP granules decreased to the levels observed in male rats, in which only a few eME granules were confirmed (Fig. 2(A)). AVP fluorescence in the pPVN was significantly decreased by OVX. AVP expression of the SON, mPVN, and PP were not significantly different between males and females of all oestrus stages as well as OVX females (Fig. 2(B),a,b,d) (Supplementary Fig. 2d). AVP expression of the pPVN showed a significant difference only in OVX females and males (Fig. 2(B),c) (Supplementary Fig. 2b,c). From these results, we determined that OVX resulted in a decrease in the AVP granules of the eME (as confirmed in females).

### High-dose oestradiol restores AVP-eGFP fluorescence in the eME

**Figure 3:**
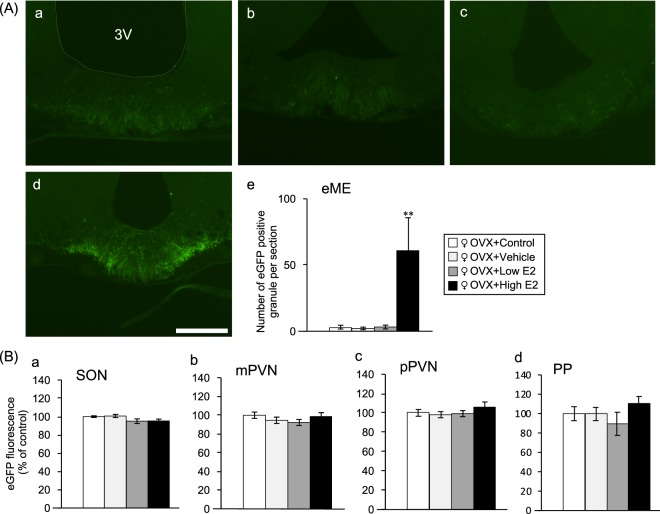
High-dose oestradiol restores AVP-eGFP fluorescence in the eME. (**A**) AVP-eGFP positive granules in the eME of the (a) control group (OVX and sham operation), (b) vehicle group (OVX and subcutaneous sesame oil), (c) low E2 group (OVX and low-dose E2), and (d) high E2 group (OVX and high-dose E2). Scale bar indicates 100 μm. (e) Number of AVP-eGFP positive granules in the eME of the high E2 (n = 7), control (n = 5), vehicle (n = 6), and low E2 (n = 7) groups. The data were presented as the mean ± SEM (one-way ANOVA) (d.f. = 3, 21; F = 33.892; **P < 0.01 compared with high E2 and other groups). (**B**) AVP expression of the (a) SON, (b) mPVN, (c) pPVN, and (d) PP in the control, vehicle, low E2, and high E2 groups. The data were presented as the mean ± SEM (one-way ANOVA).

The AVP granules in the eME of the hypothalamus were more prevalent in females but disappeared in the OVX group. Because oestrogen levels were expected to be affected by OVX, oestrogen supplementation experiments were performed (group C experiment). Only reproductive female AVP-eGFP transgenic rats were used. OVX was performed at 12 weeks, and the rats were divided into four sub-groups 2 weeks later. In the control, vehicle, and low β-oestradiol (E2) group, there was no significant change in the ME (Fig. 3(A),a–c), but in the high E2 group, AVP granules reappeared in the eME (Fig. 3(A),d). Surprisingly, it was found that when high-dose E2 was administered to the OVX rats, the AVP granules of the eME returned to a normal female state (Fig. 3(A),e). On the other hand, there was no clear difference in the SON, mPVN, pPVN, and PP of the hypothalamus. This represents the first evidence that oestrogen is associated with AVP granules of the eME. Oestrogens affected AVP granules in the eME produced by PNCs, but not AVP from MNCs.

### Relationship between oestrogen and HPA axis

**Figure 4:**
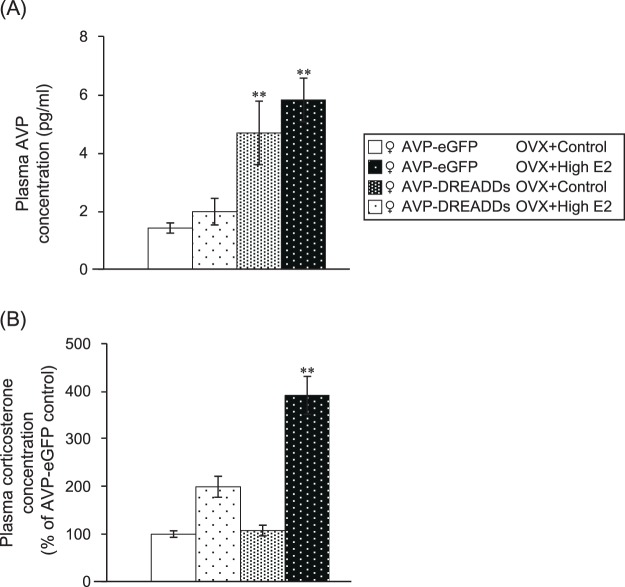
Relationship between oestrogen and the HPA axis. (**A**) Plasma AVP concentration. The data were presented as the mean ± SEM (one-way ANOVA) (n = 6 in all groups) (d.f. = 3, 20; F = 7.022; **P < 0.01 compared with AVP-eGFP and AVP-DREADDs rats). (**B**) Plasma corticosterone concentration. The data were presented as the mean ± SEM (one-way ANOVA) (n = 6 in all groups) (d.f. = 3, 20; F = 4.371; **P < 0.05 compared with each control).

The change in the AVP granules of the eME in the hypothalamus was found to be gender-dependent and oestrogen-dependent. The AVP granules of the eME were shown to be produced by PNCs, and the association between PNCs and the HPA axis was confirmed. We next investigated the effect of AVP granules in the eME on the HPA axis using the AVP-DREADDs system (group D experiment). In this experiment, we administered CNO (1 mg/kg)^13^ to all female rats (AVP-eGFP and AVP-DREADDs transgenic rats). In the AVP-DREADDs group, the plasma AVP concentrations were significantly higher than in the AVP-eGFP group. This was an effect of the DREADD system, which is influenced by AVP neurone activation. On the other hand, in AVP-eGFP and AVP-DREADDs rats, there was no significant difference in plasma AVP concentration between the control and high E2 groups (Fig. 4(A)). We ascertained that the increase in blood AVP concentration was due to the influence of magnocellular neurosecretory PVN neurones from the PP. In addition, oestrogen seems to have influenced the eME, but it did not affect the magnocellular neurosecretory PVN neurones.

The plasma corticosterone concentrations were significantly higher in AVP-DREADDs transgenic rats than in the control group when CNO was administered to the high E2 group. On the other hand, there was no significant difference among AVP-eGFP transgenic rats, even when CNO was administered to the high E2 group (Fig. 4(B)).

AVP granules accumulated in the eME when high-dose oestrogen was administered. In this high-dose state, stimulation of AVP neurones resulted in excitation of parvocellular neurosecretory PVN neurones; ACTH was stimulated by the accumulated AVP granules in the eME, and therefore the corticosterone levels significantly increased. As it has been established in past reports that parvocellular PVN neurones are involved in the HPA axis^17,18^, in this current experiment, we determined that oestrogen administration may also be involved in the HPA axis.

## Discussion

To our knowledge, this is the first study to provide evidence that gender plays a significant role in the AVP granules of the eME. Here, we also provided insight into the hypothalamic-pituitary system of reproductive male and female AVP-eGFP transgenic rats. The AVP granules of female eME were significantly more prolific than those of male eME. The AVP granules were diminished in OVX rats but recovered with high E2 administration; thus, AVP of the eME was proven to be oestrogen-dependent. In addition, when AVP neurones were stimulated by administration of CNO to AVP-DREADDs rats treated with high E2 after OVX, the plasma AVP concentrations were not significantly different, whereas the corticosterone concentrations significantly increased. This regimen triggered the HPA axis, highlighting the biological significance of the AVP granules of the eME. Therefore, our data further support theories regarding the connection between the administration of oestrogen and the stress-response effect.

AVP is mainly produced in neurosecretory neurones localised in the SON and PVN in the hypothalamus. The axons of the MNCs of the SON and PVN project into the PP, and AVP is carried by axonal transport and released into circulation. AVP secretion is mainly controlled by plasma osmotic pressure and circulating blood volume and is sensitive to the homeostasis of bodily fluids. Although there are many reports of gender differences in blood AVP concentrations, to our knowledge, there has been no report on the effects of gender regarding AVP secretion in the hypothalamus. There are reports that osmotic AVP secretion levels are gender-dependent in humans; according to previous studies, AVP secretion is suppressed in females (in comparison with males)^19^. In addition, women are affected by the oestrus cycle, and osmotic AVP secretion is suppressed from the middle stage of the luteal phase to the early follicular stages^20^. In our study, gender influenced the AVP granule count in the eME.

In another study of administration of gonadotropin-releasing hormone (GnRH) antagonist, oestrogen, and progesterone, osteotropic AVP secretion was mainly higher in the luteal phase than in the early follicular phase, primarily by the action of oestrogen^21^. The researchers found that in human women, oestrogen enhances osmoregulatory AVP secretion. However, in animal experiments, oestrogen decreased the activity of the osmotic pressure-receiving site upon osmotic pressure increase. Nonetheless, it is not known how oestrogen acts on the site and how osmotically regulated AVP secretion is accelerated. Moreover, oestrogen has been reported to enhance AVP secretion at elevated osmotic pressure but not free water clearance^20,21^. With oestrogen alone, the plasma volume increases, but the extracellular fluid volume does not increase. Therefore, the cause of the increase in plasma volume due to oestrogen is thought to be the change in the distribution of plasma and interstitial fluids in extracellular fluids^22^. In fact, oestrogen increases the amount of albumin in the blood. In our study, the osmotic AVP concentrations were somewhat higher owing to oestrogen administration, but the difference was insignificant.

AVP neurones have cell bodies in the PNCs of the PVN. They project axons to the eME, release AVP into the pituitary portal vein, and are involved in ACTH secretion from the anterior pituitary gland^19^. Various stresses including pain and inflammation stimulate PNCs of the PVN. The AVP granules of the eME influence the pituitary portal vein to the anterior pituitary gland. This promotes ACTH secretion through the V1b receptor in the anterior pituitary gland, and the HPA axis is activated by glucocorticoid release from the adrenal cortex. ACTH secretion associated with stress has a synergistic effect when combined with CRH or AVP secretion^23^. The PVN is divided into a large cellular region and a small cellular region, and CRH exists primarily inside the small cellular region. AVP coexists in CRH neurones of the PVN, and CRH and AVP granules are simultaneously secreted at the nerve terminals of the eME^24^. Unlike in rodents, the human PVN has no compartment between MCNs and PCNs, and CRH exists in PCNs^25^. Both CRH and AVP stimulate ACTH secretion from the anterior pituitary gland, and their effects are synergistic^17^. Thus, it seems convenient and practical that both peptides are secreted in a phase requiring strong ACTH secretion stimulation, but the physiological significance of the coexistence remains unclear. Previous reports showed that CRH mRNA expression increases when oestrogen is administered to OVX female rats and that corticosterone increases with chronic administration of oestrogen to OVX female rats^6–8^. In the current study, we found strong evidence linking oestrogen and AVP granules of the eME. Furthermore, the increase in AVP granules of the eME due to oestrogen-stimulated ACTH raised corticosterone levels. In the AVP-DREADDs rats, it was possible to activate the HPA axis. Therefore, we determined that oestrogen at a high concentration activates the HPA axis via AVP and modifies stress response levels.

There are reports of high oestrogen sensitivity and morphological changes in nerve endings in the ME due to aging^9^. During reproductive aging, the ME may play physiological roles because of the high oestrogen sensitivity of this region and the ability of E2 to induce dynamic morphological changes in nerve terminals and glial cells^18,26^. Previously, the morphological organisation and significant changes in cell structure of aging rat ME were reported, but short-term E2 treatment had little effect^27^. In monkeys, age-related and E2-related changes were found in the expression of genes encoding kisspeptin, neurokinin B, and prozinorphin (*KISS1*, *NKB*, and *PDYN*) in a combined dissection of the arcuate nucleus and the ME^9,28^. In our study, we examined the influence of AVP only in the eME (no AVP confirmation in the iME), but we confirmed the presence of AVP granules in the eME domain. Gender clearly played a role; AVP granules were confirmed more frequently in females. Previous reports showed that eGFP fluorescence was significantly increased 48 h after stressful conditions in the eME^26^. Therefore, AVP granule production in the eME during the oestrus cycle was considered to be delayed by 2 days. In addition, high E2 administration in rats resulted in changes in the eME.

There are several limitations in our study. OVX is surgically induced menopause, and it is not clear how this may differ from natural menopause. The effects of OVX on the hypothalamus and its complex interactions are not well established. Although a relationship between the oestrus cycle and the AVP granules of the eME was found, it is not clear why the AVP granule counts were higher in female rats. These points are to be clarified in future studies.

In conclusion, we found that gender affected AVP granules of the eME using AVP-eGFP transgenic rats. AVP granules were diminished following OVX but recovered with high E2 administration. Thus, the AVP of the eME from PNCs proved to be oestrogen-dependent. Furthermore, when AVP-DREADDs rats were administered high E2 after OVX or CNO to stimulate AVP neurones, the plasma AVP concentrations did not change, but the corticosterone concentrations significantly increased. These results suggest that stress-induced activation of the HPA axis may be caused by oestrogen-dependent upregulation of AVP from PNCs in the eME in female rats.

## Materials and Methods

### Animals

All rats were treated after 10 weeks of age once the oestrus cycle was established. Adult male and female AVP-eGFP Wistar transgenic rats, weighing 280–392 g (10–15 weeks of age), were bred and maintained as described in previous reports^11,12,29,30^. The AVP-eGFP transgenic was rat created by inserting the *GFP* gene into the *AVP* gene, allowing examination of the AVP dynamics in the hypothalamus through quantification of fluorescence intensity. Adult female AVP-DREADDs Wistar transgenic rats, weighing 320–370 g (12–15 weeks of age), were bred and maintained as described in previous reports^13^. The *AVP* gene labelled with mCherry fluorescence was modified to specifically express the DREADDs ligand. With this model, AVP neurones can be stimulated only with CNO, not endogenous ligands. In addition, GFP and mCherry fluorescence could be observed without immunostaining. All rats were housed three per plastic cage (transparent polymethylpentene, TR-TPX-200A, TOKIWA KAGAKU KIKAI, Tokyo Japan) in an air-conditioned room (22–25 °C) with a 12-h light cycle (7:00 A.M. to 7:00 P.M.) and ad libitum access to food (CLEA Rodent Diet CE-2, CLEA Japan, Tokyo Japan) and water. All experiments were performed in strict accordance with guidelines on the use and care of laboratory animals as set forth by the Physiological Society of Japan and approved by the Ethics Committee of Animal Care and Experimentation of University of Occupational and Environmental Health, Japan. All rats were screened by PCR analysis of genomic DNA extracted from rat ear biopsies^11^.

### Experimental procedure

The transgenic rats (after 10 weeks of age) were divided into four groups for experimentation (groups A, B, C, and D). Each group contained 24 to 32 rats.

The first experiment (group A) was performed to ascertain the difference in AVP between female rats (10 weeks old) undergoing a normal oestrus cycle and male rats (10 weeks old). The female AVP-eGFP transgenic rats in this first group were further divided based on the four oestrus stages (prooestrus, oestrus, metoestrus, and dioestrus)—resulting in a total of five sub-groups of males and females. Each sub-group contained four to seven rats. The oestrus cycle was confirmed with vaginal smear each morning by two researchers. Rats with irregular oestrus cycles were excluded from the experiment.

In the second group (group B), all AVP-eGFP transgenic rats were divided into six sub-groups, including the sham-operated (males and females in the prooestrus, oestrus, metoestrus, and dioestrus stage) and OVX sub-groups. Each sub-group contained four to seven rats. The rats undergoing OVX and sham operation were anaesthetised with intraperitoneal injection of a cocktail of three different anaesthetic agents (0.3 mg/kg of medetomidine, 4.0 mg/kg of midazolam, and 5.0 mg/kg of butorphanol). In the OVX sub-group, the ovaries were identified and removed, and in the sham group, the abdominal cavity was closed without treatment. OVX and sham operation were performed at the 10th week, and experiments were conducted at the 12th week.

For the third group (group C), we conducted hormone replacement experiments involving subcutaneous implantation of hormone-containing tubes in the mid-back region. We used fat-soluble E2 (β-estradiol ≥98%, Sigma-Aldrich, Tokyo, Japan) dissolved in sesame oil (Sigma-Aldrich) with Silastic tubing (1.57 mm inner diameter; 3.18 mm outer diameter; 37.0 mm in length; Dow Corning, Midland, MI, USA) filled with fat-soluble E2^31^. The rats in this group were divided into four sub-groups (five to seven rats each), in which all OVX female AVP-eGFP transgenic rats received the control (sham operation on the back), vehicle (subcutaneous sesame oil), or E2 replacement to produce low E2 (20 μg β-oestradiol/ml sesame oil) or high E2 (400 μg β-oestradiol/ml sesame oil)^31^. The low level of E2 was previously confirmed to produce a negative feedback effect on luteinizing hormone (LH) pulses but not to induce LH surges in OVX rats. High E2 levels induced LH surge and resulted in an oestrus-like state in OVX rats. We performed OVX in female AVP-eGFP transgenic rats at the 11th week, conducted hormone replacement at the 13th week, and performed experiments at the 14th week. The hormone replacement involved minimally invasive surgery for subcutaneous implantation, which has been reported to be effective for about 2 weeks^31^. We confirmed the effect of low E2 and high E2 in the vaginal smear of rats.

For the fourth group (group D), female AVP-eGFP transgenic rats and female AVP-DREADDs transgenic rats were used to measure the AVP and corticosterone concentrations via blood sampling. OVX was performed at 12 weeks of age for group D rats. These rats were further divided into four sub-groups (control group and high E2 group) at the 14th week. Each sub-group contained six rats. One week later, the plasma concentration was measured at 30 min after i.p. administration of CNO (1 mg/kg)^13^ to all rats (female AVP-eGFP and female AVP-DREADDs transgenic rats).

### Extraction of hypothalamus and pituitary gland

The male and female rats were anaesthetised with intraperitoneal injection of a cocktail of three different anaesthetic agents. The rats were perfused transcardially with 0.1 M phosphate buffer (PB) (pH 7.4) containing heparin (1,000 U/l), which were followed by 4% paraformaldehyde in 0.1 M PB. The brains and pituitaries were carefully removed, and a small block that included the hypothalamus was isolated. The blocks were post-fixed with 4% paraformaldehyde in 0.1 M PB for 48 h at 4 °C as described in previous reports^11,32^. The tissue was cryoprotected in 20% (w/v) sucrose in 0.1 M PB for 48 h at 4 °C. Fixed tissue was cut to 40-μm thickness using a microtome (REM-700; Yamato Kohki Industrial Co. Ltd, Saitama, Japan) to generate coronal sections. The sections were rinsed with 0.1 M PB and placed on glass slides. The pituitary glands were not treated.

### Confirmation of hypothalamus and pituitary gland

The locations of the SON, PVN, and ME were determined according to the coordinates in the atlas of Paxinos and Watson^16^. We isolated the pPVN from the mPVN to analyse these sections separately. In addition, the eME and iME were distinguished by labelling with an anti-OXT antibody. The sections were incubated for 48 h at 4 °C in the primary antibody solution (anti-OXT antibody raised in rabbit, AB911; Merck Corp., Darmstadt, Germany; 1:10,000). After washing twice in 0.3% Triton X-100 in PBS, floating sections were incubated for 2 h at 4 °C with a secondary antibody (Alexa Fluor 488 anti-rabbit IgG antibody raised in goat; Molecular Probes, Eugene, OR, USA; 1:2,000 in PBS containing 0.3% Triton X-100). The floating sections were placed on glass slides. We used microscopy to confirm that the sections were appropriately cut by a microtome, and we selected two sections of the SON, mPVN, pPVN, and ME per animal to observe with a fluorescence microscope (ECLIPSE E 600; Nikon Corp., Tokyo, Japan). The pituitary gland was placed on a glass slides as unprocessed and imaged twice with a fluorescence microscope.

### Evaluation of eGFP fluorescence in hypothalamus and pituitary gland

The sections containing the SON, PVN, and ME and the intact pituitary gland specimens were also examined by a fluorescence microscope (ECLIPSE E 600; Nikon Corp.) equipped with an eGFP filter (Nikon Corp.) to visualise AVP-eGFP expression. The images were captured with a digital camera (DS-Qi1Mc; Nikon Corp.). In the pituitary, eGFP fluorescence could only be observed in the PP. We averaged the value of the eGFP fluorescence intensity of these regions for each animal. The average eGFP fluorescence intensity per unit area in the SON, mPVN, pPVN, and PP was quantified with an imaging analysis system (NIS-Elements; Nikon Corp.). The fluorescent granules in the ME were quantified because measuring the eGFP fluorescence intensity of the ME is difficult, and some eGFP granules can be physically observed in the ME. The number of eGFP fluorescence-positive granules in the ME was counted manually from a photograph acquired with the same light source.

### Measurement of plasma AVP and corticosterone

At 30 min after i.p. administration of CNO (1 mg/kg), all transgenic rats were decapitated without anaesthesia. Trunk blood samples were collected into tubes (Greiner Bio-One, Kremsmünster, Austria) containing an aprotinin/ETDA mixture. The blood samples were centrifuged for 10 min at 4 °C and 1,000 *g*. After centrifugation, a 500-μl sample of plasma was collected to measure the plasma AVP concentration (SRL, Tokyo, Japan). Fifty-microlitre samples of plasma were collected to measure plasma corticosterone (Corticosterone ELISA Kit, Cayman Chemical Company, Ann Arbor, MI, USA).

### Statistical analysis

Each group of analysed rats consisted of four to seven animals. All the data points are presented as the mean ± standard error of the mean (SEM). Means were calculated from the results. Statistical significances were calculated based on one-way analysis of variance (ANOVA), which was followed by a Turkey-Kramer-type adjustment for multiple comparisons. A P-value < 0.05 was considered significant.

## Supplementary information

Supplementary info

## Data Availability

All data generated or analysed during this study are included in this published article (and its Supplementary Information files).
