# Supplementary material for: Effect of oestrogen-dependent vasopressin on HPA axis in the median eminence of female rats
Source: Sci Rep. 2019 Mar 26;9:5153. doi: 10.1038/s41598-019-41714-z (PMC6435644; doi:10.1038/s41598-019-41714-z)
Supplement: Supplementary file 1 — Supplementary info [file 41598_2019_41714_MOESM1_ESM.pdf]

Effect of oestrogen-dependent vasopressin on HPA axis in the median eminence of female rats

Kazuaki Nishimura[1,2], Kiyoshi Yoshino[2], Kenya Sanada[1], Hiroki Beppu[1], Yasuki Akiyama[1], Haruki Nishimura[1], Kentaro Tanaka[1], Satomi Sonoda[1], Hiromichi Ueno[1], Mitsuhiro Yoshimura[1], Takashi Maruyama[1], Hitoshi Ozawa[3], Yoichi Ueta[1*]

[1]Department of Physiology, School of Medicine, University of Occupational and Environmental Health, Japan
[2]Department of Obstetrics and Gynecology, School of Medicine, University of Occupational and Environmental Health, Japan
[3]Department of Anatomy and Neurobiology, Graduate School of Medicine, Nippon Medical School, Japan

Correspondence to: yoichi@med.uoeh-u.ac.jp
Yoichi Ueta, M.D., Ph.D.
Department of Physiology, School of Medicine, University of Occupational and Environmental Health, Kitakyushu 807-8555, Japan
Tel: +88-93-691-7420; Fax: +81-93-692-1711

Supplement figure 1

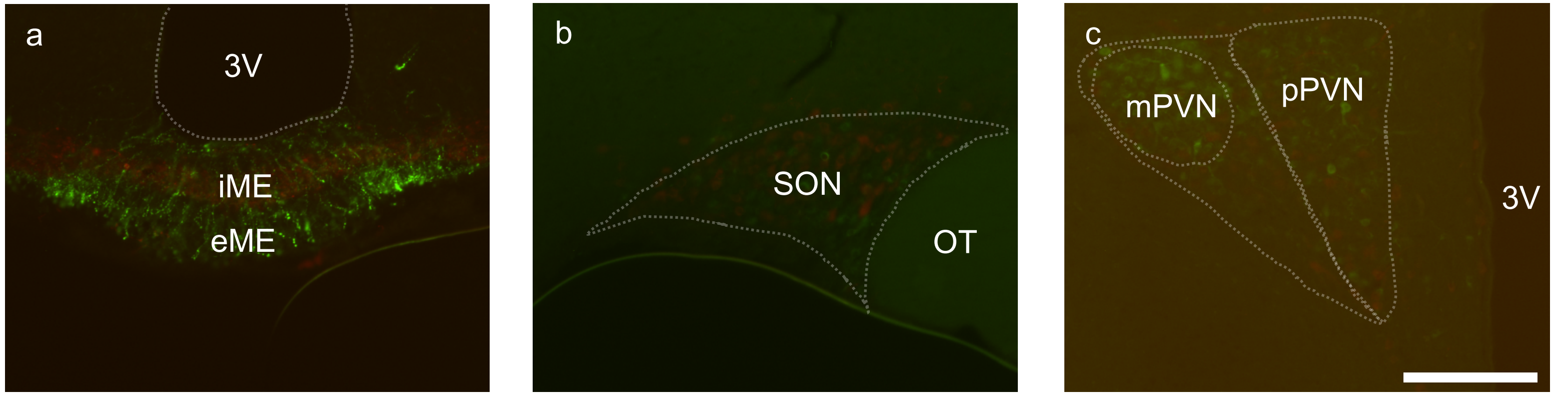

Supplementary Fig. 1 AVP-eGFP transgenic rat with anti-OXT antibody

(a) The anti-OXT antibody labelled in red was used to distinguish the internal and external layers of the ME. (b) Identification of OXT and AVP neurons in the SON. (c) The anti-OXT antibody was used to distinguish the mPVN and pPVN. Scale bar indicates 100 μm.

Supplement figure 2

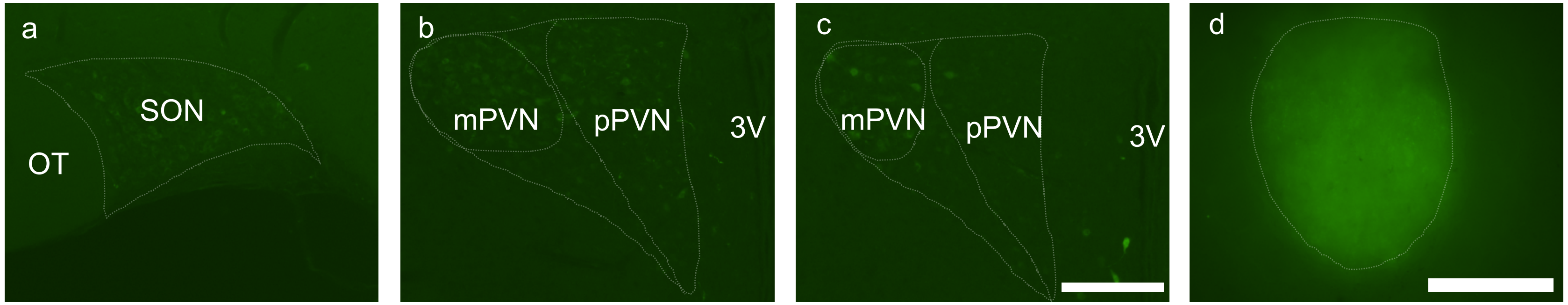

Supplementary Fig. 2 AVP-eGFP fluorescence in the SON, PVN, and PP

AVP-eGFP fluorescence was identified with a fluorescence microscope containing a GFP filter in the (a) SON and (b) PVN (mPVN and pPVN) of male and female AVP-eGFP transgenic rats and (c) PVN in the OVX group (AVP-eGFP fluorescence in the pPVN is slightly low). Scale bar indicates 100 μm. (d) AVP-eGFP fluorescence in the PP was identified in all groups. Scale bar indicates 500 μm.
